# Visualizing Extracellular Vesicles and Their Function in 3D Tumor Microenvironment Models

**DOI:** 10.3390/ijms22094784

**Published:** 2021-04-30

**Authors:** Evran E. Ural, Victoria Toomajian, Ehsanul Hoque Apu, Mladen Veletic, Ilangko Balasingham, Nureddin Ashammakhi, Masamitsu Kanada, Christopher H. Contag

**Affiliations:** 1 Institute for Quantitative Health Science and Engineering, Michigan State University, East Lansing, MI 48823, USA; evural@msu.edu (E.E.U.); toomaji2@msu.edu (V.T.); hoqueapu@msu.edu (E.H.A.); n.ashammakhi@ucla.edu (N.A.); kanadama@msu.edu (M.K.); 2 Department of Biomedical Engineering, Michigan State University, East Lansing, MI 48823, USA; 3 Intervention Centre, Oslo University Hospital, N-0027 Oslo, Norway; mladen.veletic@ntnu.no (M.V.); ilangko.balasingham@ous-research.no (I.B.); 4 Department of Electronic Systems, Norwegian University of Science and Technology, N-7491 Trondheim, Norway; 5 Department of Bioengineering, Henry Samueli School of Engineering, University of California, Los Angeles, CA 90095, USA; 6 Department of Pharmacology and Toxicology, Michigan State University, East Lansing, MI 48823, USA; 7 Department of Microbiology and Molecular Genetics, Michigan State University, East Lansing, MI 48823, USA

**Keywords:** extracellular vesicles (EVs), tumor microenvironment (TME), three-dimensional (3D) cell culture models, cell-to-matrix interactions, scaffold

## Abstract

Extracellular vesicles (EVs) are cell-derived nanostructures that mediate intercellular communication by delivering complex signals in normal tissues and cancer. The cellular coordination required for tumor development and maintenance is mediated, in part, through EV transport of molecular cargo to resident and distant cells. Most studies on EV-mediated signaling have been performed in two-dimensional (2D) monolayer cell cultures, largely because of their simplicity and high-throughput screening capacity. Three-dimensional (3D) cell cultures can be used to study cell-to-cell and cell-to-matrix interactions, enabling the study of EV-mediated cellular communication. 3D cultures may best model the role of EVs in formation of the tumor microenvironment (TME) and cancer cell-stromal interactions that sustain tumor growth. In this review, we discuss EV biology in 3D culture correlates of the TME. This includes EV communication between cell types of the TME, differences in EV biogenesis and signaling associated with differing scaffold choices and in scaffold-free 3D cultures and cultivation of the premetastatic niche. An understanding of EV biogenesis and signaling within a 3D TME will improve culture correlates of oncogenesis, enable molecular control of the TME and aid development of drug delivery tools based on EV-mediated signaling.

## 1. Introduction: EVs in the TME

Cancer cells are characterized by genetic and epigenetic alterations that provide a survival advantage, but for these cells to form a tumor, cancer cells must direct pathologic organogenesis and manage multiple interfaces with normal cells and tissues, including evasion of immune surveillance. The tumor microenvironment (TME) that is created by cancer cells is a complex network, where tumor cells communicate with stromal cells co-opted by the cancer to support tumor growth and with immune cells that are attempting to eliminate malignant growth [1]. The heterogeneous population of stromal cells and extracellular matrix (ECM) along with cancer cells comprise the TME and together coordinate the growth of a tumor. Recent studies have revealed new ways by which cells communicate with their neighboring cells through non-classical, secretory vesicles referred to as extracellular vesicles (EVs) [2]. EVs contain nucleic acids, proteins and lipids that can act over short and long-distances to mediate intercellular signaling to coordinate both physiologic tissue homeostasis and pathologic tissue states [3]. EV-mediated signaling within the TME affects each of the hallmarks of cancer and facilitates tumor growth, host cell recruitment, immunosuppression, angiogenesis, cancer cell invasion and metastasis [4,5,6,7]. In addition, several characteristics of the TME are known to affect EV biogenesis and their cargo. The implications of EV effects on the TME and the impact of EVs on tumor status necessitates the development and evaluation of clinically relevant in vitro models of human pathophysiology within the TME.

EVs are often classified according to method of biogenesis, including exosomes and microvesicles (MVs) (see Box 1). Early endosomes are endocytosed from the membrane and subsequently undergo inward budding, forming multivesicular bodies (MVBs) containing cargo packaged into intraluminal vesicles (ILVs) that are released as exosomes upon MVB fusion with the cell membrane [3]. MVs bud directly from the cell surface, resulting in a different molecular profile than exosomes. The multistep pathways of intracellular vesicle trafficking and EV biogenesis are regulated by cytoplasmic proteins such as Rab small GTPases and endosomal-sorting complexes required for transport (ESCRT) machinery [2]. MV budding is additionally regulated by the organization of phospholipids on the membrane, the contraction of cytoplasmic proteins such as actin filaments and microtubules and proteins such as the extracellular signal-regulated kinase (ERK) [8].

The biology of cancer cells is characterized by alterations in EV biogenesis and cargo loading through dysregulation of molecular regulators of intracellular vesicle trafficking. One study demonstrated that over two-thirds of tumor samples were found to express alterations in Rab gene expression, suggesting cancers modulate tumor cell secretion of EVs [9]. Many ESCRT complexes are also dysregulated in cancer, resulting in upregulated secretion of EVs and aberrant cargo loading [10]. A human polymorphism in charged multivesicular body protein 4C (CHMP4C), which tightly regulates ESCRT-III machinery, promotes genomic instability and tumorigenesis [11]. In addition, cancer cells often dysregulate endogenous levels of ceramide, a sphingolipid involved in autophagy, MVB formation and MV budding [12,13,14]. Other regulators involved in cancer exosome secretion include neutral sphyngomyelinase 2 (nSMase2) [15], phosphorylated synaptosome-associated protein 23 (SNAP23) [16,17] and Rab27A/Rab27B [18]. The dysregulation of molecular mediators of intracellular vesicle transport, fusion and fission events and EV biogenesis pathways in cancer cells partially elucidate the mechanism of increased EV secretion and abnormal cargo packaging in cancer cells that promote tumorigenesis.

Increased cellular proliferation is at the very foundation of malignancy and is associated with altered EV production and contents. The proliferation rates of cancer cells is increased by production of growth factors, upregulation of growth factor receptors or activation pathways downstream of growth factor stimulation and each of these can increase MV release, through dysregulation of MV-biogenesis via activating the small GTPases RhoA and ADP-ribosylation factor 6 (Arf6) [19,20,21]. Cancer cells overexpressing P-glycoprotein (P-gp), involved in the reprogramming of many metabolic pathways and resistance to chemotherapy, secreted EVs capable of transferring P-gp-associated multidrug resistance phenotype to surrounding drug-sensitive cancer cells [22]. The sharing of genetic, metabolic and drug resistance factors that are mediated by EVs implicate vesicular communication in the formation of the TME.

In turn and beyond the individual cancer cell, the milieu of the TME also affects EV biogenesis and cargo loading. Hypoxia occurs in regions of solid tumors that have outgrown the local vasculature and appears to increase production of exosomes via molecules such as hypoxia-inducible factor (HIF)-1*α* and Rab22A [23]. Other metabolic features of the TME that have been modeled in monolayers of cancer cells, including acidic pH, also appear to increase exosome secretion [24,25]. The tight association of oncogenesis and EV-biogenesis, the prevalence of biological effects mediated by cancer cell-derived EVs and the complexity of metabolic influences of the TME on EV production, necessitate the development and testing of clinically relevant in vitro models of human pathophysiology within the TME.

Conventional two-dimensional (2D) monolayer cultures of cancer cells have proven valuable in determining EV biogenesis and modeling tumor EV-mediated signaling. Monolayer cultures have been widely applied to the study of EV biology due to their ease of use, relatively low cost, relatively short experimental duration and high throughput. However, cells in monolayers grow with a flat morphology, demonstrate apical-basal polarity and have altered gene expression and mRNA splicing patterns [26]. As a tissue model, cells in monolayer have homogenous access to nutrients such as growth factors and disparate cell-cell and cell-matrix interactions affecting the composition and organization of the ECM including expression of integrins, proteases and chemokine receptors [27]; ECM proteins mediate many cellular functions including cell proliferation, growth, motility, apoptosis, differentiation and intracellular signal transduction. The altered ECM in 2D cultures establishes that monolayers may not accurately represent TME cell functions in vivo [28].

EV biogenesis and cargo loading is mediated by many of the biological processes that are not physiological in 2D cultures. These include cell motility [29,30], apical-basal polarity [31], differential gene expression [21,32] and growth factor receptor signaling [33]. Monolayer cultures cannot replicate the inhomogeneity of oxygen and nutrient gradients found in the TME and, thus, may not accurately represent the heterogeneous cellular gene expression profiles, signal transduction and EV populations resulting from hypoxia and hypoxia-associated conditions such as angiogenesis, necrosis and tumor acidosis [34]. All told, the pathophysiological relevance of 2D cultures in modeling EV signaling within the TME is limited and there is need for representative culture models comprised of multiple cell types and relevant physiology to examine the role of EVs in contributing to, forming, being influenced by and maintaining critical features of the TME.

Animal models are frequently used for cancer research and anti-cancer drug screening since they can provide essential information on tumor growth and tumor-host interactions within the complex TME [35]. To investigate the roles of EV-mediated signaling in cancer development, various approaches to track EVs in living animal subjects have been developed [36,37]. Even with advances in in vivo imaging that lower the cost and increase the throughput of animal studies [38], animal models are more costly and time-consuming than culture correlates and still have limited throughput; the traceability of EVs within a tumor-bearing animal over time still presents many challenges [39,40]. Xenograft models comprised of transplanted human cancer cells in immunodeficient rodents do not allow for proper tumor-stroma interactions or full vascularization and without an immune response are limited in translatability to human biology [41,42,43]. The overall average rate of a successful translation of a medicinal compound from animal models to clinical cancer trials has been reported to be less than 8% [44,45]. The use of human culture models may address this limitation and developing improved human cell culture systems with greater complexity and, hence, relevance to human cancer biology, will create opportunities in drug discovery, while simultaneously reducing study costs. Animal models will, for the foreseeable future, still be needed, but using integrated culture systems with human targets, cells and three-dimensional (3D) organ structures have become an important tool in the drug discovery process by serving as simple, fast and cost-effective alternatives to animal models [46]. New correlative culture modes of the TME will have relevance to the study of EV biology and greater understanding of EV-mediated signaling in the TME will lead to new opportunities in early diagnosis and therapy for cancer.

3D in vitro tumor models were developed by adapting several tissue engineering methods to construct cancer cell growth in 3D systems and can be designed to mimic the TME [47,48,49]. In this review, we highlight 3D culture models used to track and study the impact of EVs on the TME, determine the effects of 3D culture characteristics and composition on EV biogenesis and examine the impact of EV-mediated signaling with a 3D culture environment. Finally, we discuss novel methods to track EV-mediated systemic tumor-host interactions within the host’s tumor macroenvironment (TMaE), i.e., the interaction between the TME and the host organs and systems [50,51,52].

## 2. 3D Models of the TME

**Figure 1:**
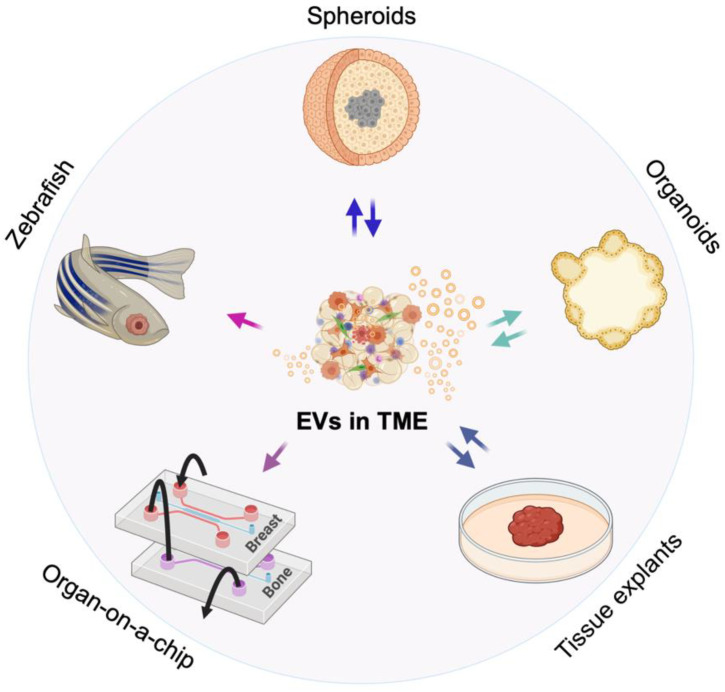
Schematic illustration of various methods to study extracellular vesicles (EVs) in the tumor microenvironment (TME) including spheroids, organoids, tumor explants, organ-on-a-chip and zebrafish. Created with BioRender.com (accessed on 3 April 2021).

Various 3D culture models have been advanced to mimic the TME and overcome the limitations of 2D cultures and the intractable nature of in vivo tumor models [35,53,54]. Such models are based on new biomaterials, 3D bioprinting of cells in matrices, organ-on-a-chip cultures and combinations that support organ-like systems, such as organoids. However, for EV research, the 3D models investigated to date that recapitulate characteristics of the TME include spheroids comprised of individual cell types, organoids and tissue explants (Figure 1) and here we address these models and their ability to mimic certain facets of EV-mediated signaling within the TME. Each system has advantages and limitations (see Table 1) and we discuss these in the context of the established and novel methods that are used to visualize EVs and assess their role in and response to the TME in each of the different model systems.

**Spheroids** are cancer cell aggregates cultured as scaffold-free anchorage-independent spheres in suspension or embedded within 3D matrices usually including only the cancer cells themselves [54,55,56]. Spheroids are formed by using the techniques of suspension, hanging drop, non-adherent surfaces, or microfluidic culture methods [56] and the nature of the spheroid is somewhat dependent on the method used [57]. Cancer cell appearance and behavior in spheroids, including motility, form and growth differ from those observed in 2D cultures [58,59]. Compared with monolayers, the 3D interactions of cancer cells cultured in spheroids better mimic the spatial and physical aspects of the TME while retaining the simplicity of culture setup, feasibility and high-throughput capacity [46]. Matrix interactions can surround cells in spheroids and better recapitulate physiological characteristics such as tissue-specific cell polarization, matrix synthesis and remodeling and differential gene expression. EVs have been isolated from spheroid-conditioned media using a variety of methods [60] and characterized according to the minimal information for studies of EVs (MISEV) 2018 guidelines [61].

3D culture conditions affect EV biogenesis, size and cargo [62]. For instance, human gastric cancer spheroids cultured in an agarose microwell array [63] secrete EVs at an increased rate and of a smaller size than those from the same cell lines in 2D cultures [64]. The spheroid-derived EVs appear to be more representative of those derived from patient plasma [65]. Likewise, murine colorectal cancer (CRC) spheroids also secreted an increased number of EVs per cell compared to parental cells cultured in monolayers [66]. Hypoxic conditions also increase the rate of biogenesis of tumor cell-derived EVs that promote tumor cell invasion and metastasis [67], inflammation [68], a pro-tumorigenic phenotype in mesenchymal stem cells (MSCs) and angiogenesis [69]. Cancer spheroids imitate spatiotemporal oxygen and nutrient gradients, resulting in hypoxic and necrotic areas that closely mimic tumor gene expression profiles observed in tumors grown in vivo [34].

Co-culturing tumor cells with stromal cells in a 3D environment [70,71] permits continuous EV-mediated crosstalk to better replicate induction of pro- or anti-tumorigenic cellular activity in processes such as angiogenesis [72] and immune cell evasion [70,71]. Spheroid models can be initiated with and comprised of, in part, cancer stem cells (CSCs), that provide CSC-derived EVs that aid tumor progression and chemoresistance [73]. Mechanical cues from soft 3D fibrin gels were found to promote the growth and selection of CSCs in spheroid cultures [74] reminiscent of the colony-forming unit assays that are performed by growing stem cells under soft agar. Moreover, these CSCs, as well as their EVs, were characterized by distinct physical properties, relative to their differentiated parental cells and their EVs [75]. This included a more compressible, or softer, phenotype. These more compressible EVs showed improved drug delivery in vivo.

**Organoids** are 3D cellular aggregates derived initially primarily from stem cells that retain certain functionalities of their respective organs of origin and can phenocopy organotypic differentiation and cell-type composition, with the ability to self-organize and propagate through self-renewal [76,77,78]. Organoids are typically differentiated from induced pluripotent stem cells (iPSCs), or embryonic stem cells (ESCs) cultured in growth factor-supplemented media purposed to differentiate cells through a germ layer to a terminally differentiated miniatured organ type. However, this definition is often inconsistently utilized in the field and so we will define each unique model in an attempt to standardize terminology. Organoid-derived EVs, like all EVs, should be isolated, defined and characterized according to the MISEV 2018 guidelines for consistency and uniformity in the field [61].

Although stem cell-derived organoids require more expensive reagents and culture time than spheroids, they offer high throughput experimental parameters and provide a physiologically relevant platform, prompting cells to produce EVs that are more representative of a patient plasma EVs in size, number and cargo contents [62]. Since organoids maintain almost all varieties of an organ’s cells, the EVs released are heterogeneous and more representative of human physiology [31]. The tissue architecture in organoids can be remarkably similar to organs and tissues, allowing for a more accurate representation of full tissue-specific tumorigenesis. Differentiating organoids from a stem cell population supports the long-term culture of normal tissue-matched cells expressing varying degrees of stemness and multilineage differentiation. This is particularly advantageous in modeling the role of EVs in transforming a normal differentiated cell or stem cell into a tumor cell or CSC, respectively. Furthermore, organoid ability to replicate the microanatomy of organ-specific stem cell niches opens gateways to research EV communication within the stem cell microenvironment along with the TME. Since cancers must, by definition, be derived from a cancer initiating cell(s) and cancer initiating cells are characterized as having stem-like features, these cultures are useful in studying the role of EVs in oncogenesis and the progressive steps required to achieve the hallmarks of cancer and establish malignancies.

**Tissue explants** are tissue fragments from a patient or animal that retain the histological features of the original tissue or tumor [79], which allows for the TME to be preserved along with the heterogeneity of cell types within a tumor site and the differences between patients. Several culture methods have been described for explants and include submerging the tissue in media, using a grid to keep fragments in contact with the media, or culturing fragments on gelatin or collagen sponges sitting in media. The duration of tissue explant cultures is relatively short to ensure cellular viability and retain the characteristics of the tissue. The original tissue architecture in the explants tend to break down at three days in culture due to adaptation of the tissue explants to the culture environment [79]. A recent study demonstrated that ex-vivo culture of freshly resected breast and prostate tumor specimens, termed patient-derived tissue explants (PDEs), could be cultured for up to six days on a gelatin sponge without losing the native tissue architecture, microenvironment, cell viability and key oncogenic drivers [80]. Tissue explants offer some advantages in being relatively inexpensive compared to mouse models, preserving the original tissue architecture and containing normal, nearly healthy tissue from the surrounding area as an internal control [79]. EVs can be enriched and collected from the conditioned media of tissue explant cultures using routine isolating methods, such as ultracentrifugation [81]; however, it must be noted that in these models the EVs are derived from a variety of cell types and markers of cellular origin need to be used to study specific populations.

## 3. Advances in Visualizing EVs in 3D Models and Assessing Their Role

Conventional EV labeling methods have mainly relied on lipophilic fluorescent dyes such as PKH, DiR and DiI, enabling visualization using a variety of fluorescence microscopy tools. Alternatives to dye labeling methods include genetic modification of cells to express fluorescent or bioluminescent proteins fused with a transmembrane domain of a membrane protein, EV-associated protein, or a membrane-anchoring signal sequence [36]. Small ATP-independent luciferases such as *Gaussia* luciferase (Gluc) and NanoLuc have been used for tracking EVs in culture and living subjects [82,83,84,85]. Since EVs generally lack ATP and extracellular levels of ATP are negligible, the development of these bioluminescent reporters was critical to enabling bioluminescent imaging approaches, which have led to rapid and high-throughput EV screens [86]. As living biomarkers, bioluminescent reporters are well-suited for the study of complex, multidimensional living systems comprised of multiple cell types [38]; however, given that they are a relatively weak biological source of light that cannot be readily controlled, bioluminescent reporters lack the ability for high resolution imaging.

Dynamic imaging of living cells in culture offers high-resolution dynamic data that cannot be obtained in living subjects and, thus, can lead to unique discoveries. For example, Chen et al. reported on the mechanism of how EVs can cross the highly selective semipermeable endothelial cell border of the blood–brain barrier (BBB) using culture correlates [87]. EVs stably expressing Gluc and green fluorescent protein (GFP) were used to assess EVs ability to cross the BBB in an inflammatory model utilizing brain microvascular endothelial cells cultured on a collagen type I-coated Transwell dish. They determined that EVs only crossed the BBB when it was treated with the pro-inflammatory cytokine, tumor necrosis factor alpha (TNF-*α*) and resulted from active endocytosis, specifically clathrin-dependent and caveolae-dependent endocytosis.

In vivo bioluminescent imaging (BLI) can employ similar reporters as seen in a study by Lai et al., where a membrane-bound Gluc was fused with a biotin acceptor peptide to allow both to be expressed on engineered cell membranes and on EV membranes [82]. Gluc on the surface of EVs allowed for BLI and determination of EV biodistribution in a mouse model and the biotin acceptor peptides on the EV surface allowed for labeling with streptavidin-Alexa680 for fluorescence imaging. The primary absorbers of short wavelengths of visible light (UV to blue) in living tissues are hemoglobin and melanin and all ATP-independent luciferases characterized to date are blue-emitters. Absorbance is reduced when using luciferases with longer wavelengths of emission, but these tend to be larger molecules and require ATP; some of the recently described luciferases (e.g., AkaLuc) emitting at peaks above 600 nm (AkaLuc has a *λ*_max_ of 677 nm). Continued development of new reporter proteins with unique characteristics will enable new imaging strategies for the study EVs.

There is a need to study EVs in the TME where the contextual influences of the tissue microenvironment are intact; several approaches have been developed for this purpose. A recently developed imaging technique for tracking EVs in tissue explants allowed for characterization of localized EV contents without requiring tissue dissociation or EV isolation, i.e., in situ [88]. Tissue explant fixation with formalin and 1-ethyl-3-(3-dimethylaminopropyl) carbodiimide fixes cells and EVs, allowing for staining and characterization [88]. In bovine vitreous humor tissue samples, RNA and protein were observed colocalized in the extracellular space, in structures similar to that characteristic of EVs. In a murine mammary tumor tissue explant, extracellular DNA was observed in vesicles along with protein and RNA [88] and a higher number of EVs were observed in mammary tumor tissue explants as compared to normal mammary tissue explants. Tools such as these are serving to localize EVs in the TME and suggesting a key role of EVs in biology given the observation of EV composition in different TME configurations.

Labeling EVs with dyes or through expressing anchored fluorescent proteins can be useful, but these approaches have some drawbacks including stability and specificity as well as the possibility of interfering with the biological function of EVs by altering their surface. Raman microscopy, a technique where the signal depends on chemical bonds present in a system, may be used to image EVs. The Raman signals from EVs will be similar to those from cells given the similarity of EV composition to cellular composition and, therefore, to differentiate EVs from surrounding cells by Raman imaging, tags such as alkynes or carbon-deutrium that have unique Raman signatures may be used to label the EVs [89]. The Raman signals from these tags do not match anything occurring endogenously in living organisms, so it is easy to distinguish them from the surrounding biological system. Since these molecular tags are smaller than fluorescent dyes, it is possible that they would not impair the natural function of EVs as much. In addition, Raman imaging is performed at a single long excitation wavelength, usually at 785 nm which penetrates deeply into mammalian tissue, resulting in multiple emission wavelengths from the different molecular bonds such that multiplexing is easily performed.

Horgan et al. labeled EVs from breast cancer cells and healthy breast tissue cells using deuterium oxide (D_2_O). Volumetric Raman imaging was then used to create 3D images of cells treated with D_2_O-labeled EVs and the multiplexing revealed subcellular components, serving as fiducials for determining EV location within cells. In this manner D_2_O-labeled EVs could be tracked in breast cancer and healthy breast epithelial cells with comparisons at 37 °C and 4 °C to reveal the energetics of EV uptake. The use of Raman tags to label EVs in conjunction with Raman spectroscopic imaging as well as automatic image processing allows for the imaging and molecular characterization of EVs and surrounding cellular components. It is worth noting that the data acquisition in current systems for Raman spectroscopy at high resolution is not fast enough to image live cells, so only fixed cells were used for this study [89]. Surface-enhanced Raman (SERs) particles, comprised of a metal, a Raman active substance and a silica coating increase signal-to-noise ratios (SNR) and can shorten data acquisition time [90], but in their current form may not have applicability to the tracking of individual EVs given their relatively large size. Raman-based techniques may be applied to the study of EVs in 3D cultures or tissue explants after fixing.

A clinical EV imaging approach recently developed is a label-free multimodal multiphoton imaging technique to characterize EVs in live tissues [91,92]. Sun et al. used a combination of four nonlinear optical imaging modalities to visualize the intrinsic optical properties (autofluorescence) of collagen fiber reorganization, elastin fibers, flavin adenine dinucleotide (FAD) in cell cytoplasm, lipid-water interface and nicotinamide adenine dinucleotide (phosphate) [NAD(P)H] of EVs. This allowed for generating a map of the TME to visualize EV locations relative to the biological context of cancer cells. Tissue explants of a breast tumor and healthy breast tissue were examined with this multimodal optical imaging system and the images were validated with histology staining. EVs were visualized using the third-harmonic (THG) imaging mode, which highlights interface surfaces as EVs have higher surface to volume ratios. The EV density was much higher in the breast cancer tissue explants. Characterization of EV density and distribution revealed that the density of EVs in the breast cancer tissue was correlated to the pathology of the tumor and during later stages of tumor invasion the EV density increases with most EVs found in desmoplastic regions, sites of fibrosis surrounding a tumor that is broken down by tumor cells to allow them to guide migration, potentially showing an increase in intercellular communication in this area by way of EVs. Data acquisition times when using intrinsic optical (autofluorescent) properties of tissues are long because the signals are weak, similar to Raman imaging of intrinsic optical properties.

The advantage of imaging intrinsic optical properties of tissues is that it is label-free and when applied to EVs derived from breast cancer cells and tissues, You and colleagues noted differences between EVs from normal and malignant tissues [92]. In addition to THG signals from collagen, 2 photon-fluorescence (2PF) of FAD and 3-photon fluorescence (3PF) of NAD(P)H were used to evaluate EVs isolated from two human breast cancer lines and a healthy human breast line [92]. Cancer-derived EVs showed higher 3PF intensity than healthy EVs, which was shown to be due to an enrichment of NAD(P)H. This difference was also present in EVs in tissue explants from patients with invasive ductal carcinoma and healthy donors. The levels of NAD(P)H in the tissue explant EVs were also dependent on the stage of cancer. These studies emphasize the important role of imaging intrinsic optical properties for metabolic profiling of EV since this approach has potential clinical applications for diagnostics.

Box 1 **Subtypes of EVs.**

Extracellular particles include EVs and exomeres [93]. EVs have been historically classified based on cell origin and biological function, or by pathway of biogenesis [2,94]. Exosomes, ranging near 40–120 nm in diameter, are secreted upon fusion of MVBs within the cell with its membrane, releasing the vesicles. MVs, ranging near 50–1000 nm in diameter, are formed from plasma membrane budding directly from the cell surface. Apoptotic bodies, that can be anywhere from 500–2000 nm in diameter, are fragments of cells undergoing apoptosis [95].

Each of these EV subtypes may have distinct biological cargo and signaling effects [3,96,97]. However, due to the challenges of purifying different EV populations and exomeres, researchers have recently begun to classify extracellular particles based on size. Exomeres are typically <50 nm in diameter, small EVs are <200 nm in diameter and medium and large EVs are >200 nm in diameter [61,93].

In this review, we will refer to EVs as comprising exosomes and MVs. Alternatively, we may use either of these classification types, following what the original authors used in their research articles.

## 4. Effects of Scaffolding in 3D Cultures on EV Biogenesis

Despite limitations, monolayer cultures still have a place in the study of mammalian biology. However, in the body, cells live within the surrounding ECM, which provides essential biochemical signals and mechanical cues to the cells and it is therefore necessary to create environments that mimic the native tissue as closely as possible by selecting relevant biocompatible scaffolds [98]. Scaffold material choices may include metals, glasses, biological or synthetic polymers and ceramics [99]. These materials can provide the scaffolds to create 3D structures; selection of materials will depend on possible fabrication methods, desired scaffold structures with features on the micron or nanometer scales. Nanoscale features are needed for determining cellular responses to the scaffold, including cell adhesion, morphology, differentiation, and apoptosis.

Recent studies have shown that extracellular signals of the biomaterial change cellular biochemical activities and genetic programs, which also influence the biogenesis of EVs [65]. EVs derived from Ewing’s sarcoma (ES) cells in a type I collagen-hyaluronic acid (HA) 3D scaffold were markedly smaller than those obtained from 2D cultures. EVs isolated from the engineered tumor model had a similar size distribution to those circulating in the plasma of ES patients. By contrast, growing ES cells as 3D cell aggregates on a flat plastic surface or in type I collagen-HA 2D cultures did not result in similar EVs. Higher levels of mRNA encoding histone methyltransferase enhancer of zeste 2 (EZH2), an important mediator of ES tumor growth and progression [100], were detected in EVs derived from engineered tumor models compared to those obtained from 2D cultures; this also corresponds to what was observed in the plasma of ES patients [65]. These EVs from the engineered tumors were shown to transfer cargo to bone-derived MSCs, as assessed by measuring EZH2 mRNA in the recipient cells and by RNA staining of the EVs. This study indicates that both appropriate ECM proteins and 3D architectures are essential for the observed effects of the TME on EV size and cargo. Interestingly, an exosome marker CD81 was only detected in EVs in 2D cultures but not in the tissue-engineered 3D culture and the tension forces among the tumor cells appear to be related to this change in EV biogenesis [65].

When EVs derived from human cervical cancer HeLa cells in 2D and 3D cultures, formed in a self-assembling peptide-based hydrogel [101], were compared to EVs from cervical cancer patient plasma [102], EVs derived from the HeLa spheroids showed a more uniform distribution of size. In contrast, EVs from HeLa cells grown in monolayers showed a broad range of size distribution. To achieve the same cell number as in a 48 h old 2D culture, the 3D HeLa culture required 11 days. At this time, the 3D cultured cells released more EVs per cell. Importantly, the profiles of small noncoding RNAs from EVs derived from the 3D HeLa spheroids exhibited greater similarity (~96%) to EVs derived from plasma from cervical cancer patients [102]. In a similar study, EVs from CRC patient-derived organoids embedded in Matrigel were analyzed. In this study, EVs with the marker CD63 predominated in EVs from CRC cell line-derived 3D cultures, whereas EVs with the marker CD81 were dominant among EVs from CRC patient-derived organoids [103]. Of note, *APC* gene mutation and collagen deposition were identified as critical factors for increasing EV release from tumors in this study of colorectal cancer, detected by measuring the amount of CD81 signal using a bead-capture assay. ECM selection determines many characteristics of EVs and this can in turn modify the ECM.

**Figure 2:**
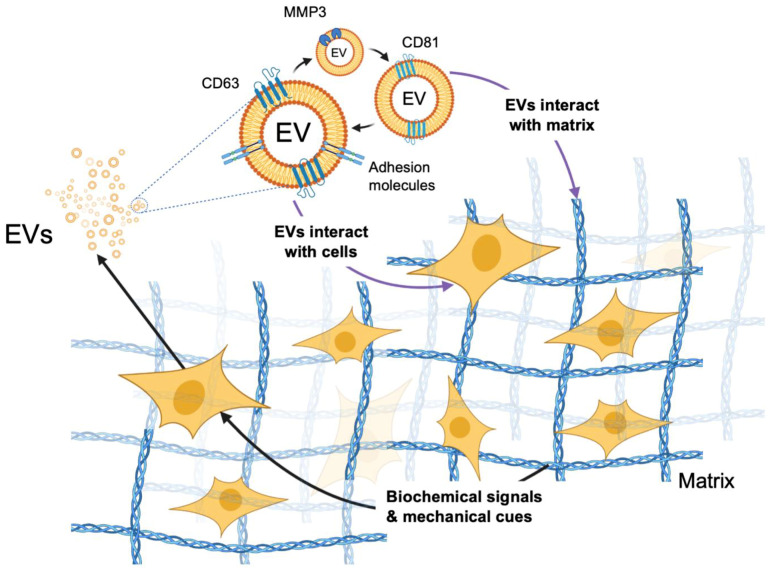
Schematic illustration of tumor microenvironment (TME) showing how the extracellular vesicles (EVs) are secreted by tumor cells that act on other cells in the tumor and on the extracellular matrix (ECM). In the TME, interaction with the ECM also influences tumor cells and lead to EV release. ECM sends biochemical signals and mechanical cues to tumor cells that affects EV biogenesis and cargo loading. Created with BioRender.com (accessed on 3 April 2021).

Not only does the ECM determine molecular composition of EVs, but also cancer cell-derived EVs have been shown to modify the ECM composition. This occurs either directly by the enrichment of proteolytic enzymes in EVs or indirectly by regulating the ability of target cells to synthesize or degrade matrix molecules, as depicted in Figure 2 [104]. The diffusion and transportation processes of EVs through the ECM were recently studied using a decellularized native ECM from lung tissue and alginate hydrogels [105,106]. EVs were isolated from mouse MSCs expressing an exosome marker CD63 fused with Katushka2S, a far-red fluorescent protein [107]. High-speed 3D microscopy with deconvolution was employed to calculate their displacement at different conditions. Interestingly, despite the average pore size of the matrix being under 10 nm, half of the introduced EVs escaped from the ECM after 24 h, indicating that EVs (50–150 nm in diameter) can travel through these dense structures. In addition, EVs in stiff stress-relaxing hydrogels were released more rapidly than EVs in soft stress-relaxing matrices. By contrast, the release of polystyrene nanoparticles and liposomes with similar sizes was minimal and did not change with different mechanical properties of the hydrogels.

Along with modifying the ECM composition, EV delivery to tumor cells in scaffolded 3D cultures has also been shown to affect proliferation in a more physiologically relevant manner than 2D monolayers. This was demonstrated by anticancer treatment using genetically engineered HEK293T cell-derived EVs containing inactive cytosine deaminase fused with uracil phosphoribosyl transferase (CD-UPRT) administered to glioblastoma cells cultured in 2D monolayers, 3D spheroids and a xenograft glioblastoma mouse model [108]. Subsequent treatment with the prodrug 5-fluorocytosine (5-FC) activated CD-UPRT to induce inhibition of DNA replication and apoptosis. EV treatment of tumor spheroids grown in 0.3% methylcellulose matrix reduced cell viability and significantly decreased spheroidal volumes (size) without changing the number of spheroids formed, implying the EV therapeutic had a larger impact on proliferation than cell adherence. Engineered EV and prodrug treatment of 2D monolayer cultures decreased cell viability by 30%, whereas treatment of mice induced a 70% reduction in tumor growth more similar to 3D spheroid treatment effects [108]. Thus, 3D scaffolded cultures affect many characteristics of EV signaling within the TME including EV biogenesis and cargo loading, ECM composition and rate of cell proliferation.

Scaffold-free spheroid fabrication methods do not use biomaterial matrices [55]. The influence of adhesion independent 3D cellular architecture on the release and cargo of EVs derived from gastric cancer cells was studied using an agarose microwell array, which allowed long-term culture of spheroids [63,64]. 3D cultures were significantly more efficient in producing EVs than 2D cultures, while the mean diameters of EVs isolated from spheroids were smaller than that of cells in 2D cultures. The profiles of small noncoding RNA present in EVs produced from cells in 2D and 3D cultures were similar, but specific microRNA (miRNA) signatures were distinct. Moreover, proteins associated with the small GTPase Arf6 pathway [64], which regulates the shedding of EVs at the plasma membrane [19], were significantly reduced. EVs derived from cells in 3D cultures showed global upregulation of miRNA and downregulation of proteins [64]. Association of 3D culture- and 2D culture-derived EVs with recipient cells was confirmed by labeling the EVs with fluorescent dye PKH26, followed by flow cytometric analysis. EVs derived from human gastric cancer MKN45 cells grown in 3D cultures significantly increased invasion of recipient cells compared to those in 2D cultures; this functional difference appeared to be cell type-dependent [64].

## 5. EV-Mediated Signaling within Models of the TME

### 5.1. Monitoring EVs and Their Function in Spheroids and Organoids

Cancer spheroids cultured in the absence of a scaffold form natural cell-to-cell and cell-to-matrix interactions that affect cellular polarity and organization and EV cargo; cellular polarity is commonly disrupted in carcinomas and this can affect EV cargo packaging. Human colon carcinoma LIM1863 cells grow as free-floating multicellular spheres of polarized cells around a central lumen with crypt-like structures containing columnar and goblet cells [31]. These colonic spheroids release two distinct populations of EVs based on cell polarity. EVs containing epithelial tight junction glycoprotein EpCAM are apically released, whereas EVs containing colon epithelial cell-specific transmembrane glycoprotein A33 are basolaterally secreted [31]. The 3D spheroid environment can influence EV cargo to have an affect on cell-to-matrix interactions and EVs carry matrix metalloproteinases (MMPs), which are a family of proteolytic enzymes that are used by cancer cells to digest the ECM [109]. Some members of MMPs are highly expressed in colon adenocarcinoma cells. Gene knockouts (KO) of one of these MMPs (MMP3) in LuM1 cells demonstrated a decrease in the EV markers tetraspanins CD9 and CD63 when the cells were grown as spheroids. This KO also correlated with destabilized EV membrane integrity and smaller spheroid size with larger necrotic areas. The treatment of MMP3-KO spheroids with EVs produced by spheroids expressing MMP3 rescued the proliferation of MMP3-KO spheroids and the expression of MMP3 and CD9. The uptake of EVs by spheroids was tracked by expressing palmitoylated-fluorescent proteins in EV donor cells. The cell-to-cell and cell-to-matrix interactions of a 3D microenvironment can affect EV secretion and cargo loading, providing more representative information about EV populations and their signaling effects within the TME. In addition to gene knockout experiments, chemical modification of EV function was demonstrated in 3D culture correlates of the TME.

Breast cancer cell-derived EVs were used to test the effects of peroxisome proliferator-activated receptor-*γ* (PPAR*γ*) and retinoid X receptor (RXR) agonists on breast cancer spheroids [68]. PPAR*γ* agonists have anti-inflammatory activity and inhibit mammosphere formation by binding to DNA as heterodimers with RXRs. PPAR*γ* regulatory miRNAs were found in higher amounts in hypoxic MCF7-derived EVs compared to normoxic MCF7-derived EVs. Hypoxic cancer cell-derived EVs only slightly increased MCF7 spheroid formation, but induced the expression of a stem cell regulatory gene, Notch3. Treating the EV-donor MCF7 cells with PPAR*γ* and RXR agonists resulted in EVs that reduced MCF7 spheroid formation and Notch3 expression. This indicates that the PPAR*γ* and RXR agonists can affect EV-mediated cross talk in breast cancer [68].

Spheroids may also be used to test EVs as drug delivery vehicles [110]. EVs from fibroblasts were manipulated to carry the anti-cancer drug, methotrexate (MTX) and engineered with a proapoptotic glioblastoma multiforme (GBM)-targeted peptide KLA-LDL. The engineered EVs were labeled with PKH26 fluorescent dyes for monitoring cellular uptake by U87 GBM cells in 2D culture and spheroids. These EVs were able to penetrate deeper into the spheroid than EVs without the targeted peptide, indicating that the KLA-LDL peptide may allow the EVs to penetrate deeper into a tumor, an insight that could not be gleaned from 2D culture models. These EVs decreased the growth of the GBM spheroids over time. The targeting and anti-tumor effects of the engineered EVs were also confirmed in a GBM mouse model. Spheroids provide a better understanding of tumor penetration of therapeutic EVs, allowing for better future EV engineering.

### 5.2. Cancer Stem Cells (CSCs) in 3D Cultures

CSCs can regenerate the organization and cell types in a tumor and are often cultivatable as spheres in vitro [111,112]. CSCs exhibit many defining characteristics such as self-renewal, continuous proliferation capacity, tumor initiation and progression, a relatively slow cell cycle and entrance into dormancy, chemoresistance, expression of stem cell markers and pluripotency [113]. Mouse Lewis lung carcinoma (LLC) cell-derived EVs induced CSC properties in differentiating murine induced pluripotent stem cells (miPSCs) as indicated by GFP expression under the Nanog promoter [114]. When grown in suspension culture, the EV-treated miPSCs formed spheroids that expressed higher levels of the Yamanaka factors Sox2 and Klf4, as compared to the same treatment of cells grown in adherent monolayer cultures. This implies a combination of EV-mediated signaling and 3D culture-induced cell interactions can potentially preserve some stemness in CSCs [114]. Human induced pluripotent stem cell (hiPSC)-derived EVs were also shown to increase cellular proliferation, growth and viability in human cortical spheroids [115]. Intestinal fibroblast-derived EVs carry amphiregulin, a membrane-bound member of the epidermal growth factor (EGF) family. These EVs have been shown to maintain small and large intestinal stem cell populations in 3D intestinal organoids by transferring Wnt and EGF activity. Thus, intestinal fibroblast-derived EVs can maintain a model intestinal stem cell niche, which is often involved in tumor progression and therapeutic resistance [116]. These examples demonstrate the potentially high impact of stem cell- and CSC-derived EVs in the TME.

Culturing tumor cells in stem cell medium often results in characteristics of a 3D microenvironment including heterogeneous cell populations. These media also induce certain cell populations to express CSC markers and/or exhibit chemoresistance. For instance, human prostate neuroendocrine adenocarcinoma PC-3 cells cultured with stem cell medium in 3D cell culture plates formed large grape-like aggregates with more intercellular space, fewer areas of intercellular adhesion, a reduced cell proliferation rate and cell differentiation [117]. Compared with 2D cultures or spheroids in serum-containing medium, the PC-3 cell aggregates in stem cell medium formed larger cellular aggregations with increased intercellular adhesion, expression of stem cell markers and oncogenes, increased hypoxia levels and slower cellular proliferation rate, better representing in vivo tumor status. Notably, this 3D environment promoted the secretion of EVs carrying epithelial cell adhesion molecule (EpCAM), a marker of CSCs, as well as CD9.

CSC-derived EVs play a significant role in chemoresistance by recruiting immune cells and inducing a pro-tumor phenotype. When cultured in stem cell medium, murine CRC CT26 cells grow as CSCs in spheroids (colonospheres) that exhibit increased chemoresistance and orthotopic tumorigenicity [66]. CT26 cells expressing GFP-Luciferase were used for in vivo tracking of EVs and an elevated luciferase activity was detected in bone marrow cells of mice bearing colonosphere-derived tumors expressing GFP-Luciferase. Moreover, colonosphere-derived EVs containing FLAG-tagged CD81 were intravenously injected into tumor-free mice and CD11b^+^/Gr-1^+^ neutrophils were identified as the predominant group engulfing exogenous tumor EVs in the bone marrow. Repeated doses of colonosphere-derived EVs into tumor-free mice also increased the number of neutrophils present in the bone marrow and circulation, as well as the primary tumor because cells in the colonospheres increased expression of the neutrophil-recruiting chemokines CXCL1 and CXCL2. In addition, EVs derived from CSC-rich colonospheres were found to contain 5′-triphosphate RNA that activates NF-kB in neutrophils and increase interleukin-1 beta (IL-1*β*) secretion, leading to prolonged neutrophil survival as well as promoted tumor cell survival in the primary TME and peritoneal spreading of tumor cells in vivo. CRC patient data suggest that this is associated with overexpression of epithelial to mesenchymal transition (EMT) regulator Snail and interleukin-8 (IL-8) in CSCs in the TME [66].

The heterogeneous cell populations found within GBM contains subpopulations of stem cell-like tumor cells supported by tumor vasculature. GBM cells downregulate the tumor suppressor microRNA, miR-1. This miRNA directly targets the EV protein annexin A2 (ANXA2), a pro-oncogenic factor in GBM known to promote proliferation, invasiveness and angiogenesis [118]. The cells cultured in stem-like conditions formed spheroids, termed stem-like neurospheres, with cells and EVs containing decreased levels of miR-1 and increased ANXA2. GBM neurosphere-secreted EVs promoted tumor cell migration, sphere formation and angiogenesis. Interestingly, miR-1 overexpression in neurospheres increased EV-mediated transfer of miR-1 and downregulated ANXA2 levels in recipient cells and diminished EV-mediated pro-tumorigenic effects [118]. Furthermore, miR-1 expression decreased GBM-derived EV size and increased levels of small RNA cargo (<40 nt) without altering the CD63 expression level. Associated with worse patient outcomes, increased ANXA2 expression was confirmed to be inversely correlated with miR-1 expression in GBM patient samples [118].

The interactions between CSCs and surrounding stromal cells mediate tumor cell growth and survival. These interactions are mediated by CSC-derived EVs capable of recruiting distant cells such as MSCs to the tumor. Renal CSCs, which are able to grow in spheres and initiate tumors in vivo, increased bone marrow-derived MSC chemo-attraction and migration, potentiating a role for these EVs in promoting MSC recruitment from the bone marrow [119]. In response, CSC EV-stimulated MSCs promoted angiogenesis and tumor cell migration and secreted pro-inflammatory cytokines. These CSC EV-treated MSCs also enhanced tumor growth in vivo [119]. The significance of these observations is that EVs may play a key role in recruiting stromal cells to the TME, contributing to tumor progression and maintenance.

Human high-grade glioma tissue samples cultured in stem cell-enriching medium grow as tumor spheroids rich in two distinct subpopulations of glioma stem-like cells (GSCs)—proneural and mesenchymal—with proteome analyses recapitulated differential protein expression patterns among the cell subtypes [120]. Spheroids containing co-cultures of mesenchymal and proneural GSCs showed dynamic EV-mediated crosstalk between cell subtypes. Mesenchymal GSC-derived EVs increased proneural GSC sphere frequency, number, viability and volume potentially due to increased phosphorylation of critical pro-oncogenic kinases or EV-mediated transfer of epidermal growth factor receptor (EGFR). Data analysis obtained from The Cancer Genome Atlas (TCGA) showed that a mesenchymal GSC EV protein signature was associated with a better prognosis in patients with proneural GSC tumors and proneural GSC EV protein signature was associated with a worse prognosis in patients with mesenchymal GSC tumors. This suggests that EV communication among heterogeneous cell populations of the TME may lead to decreased survival. Similarly, patient-derived GSCs cultured in spheroids ex-vivo were found to secrete EVs that contain vascular endothelial growth factor A (VEGF-A), inducing angiogenesis in vitro [121]. EVs likely play a role in disease progression and this can be modeled in culture correlates of cancer.

The extent of cellular stemness in 3D cultures changes the tumor biology and EV production in ways that may predict tumor growth in vivo. Colon adenocarcinoma spheroids grown in 3D cell culture with stemness-enhancing medium showed an elevated expression of ATP-binding cassette transporter G1 (ABCG1), which is a cholesterol lipid efflux pump, at levels higher than spheroids grown in serum alone [122]; stemness-enhanced spheroids showed a decrease in cell proliferation rate, as indicated by Ki67 staining and an increase in EV production compared to serum-stimulated spheroids. An increased expression of ABCG1 was found in cells on the periphery of the spheroids and EVs derived from these spheroids did not contain ABCG1, suggesting that ABCG1 plays a role in transporting substances into and out of the spheroid but not in EV to cell communications. Knocking down ABCG1 in spheroids resulted in an intracellular accumulation of EVs and decreased spheroid growth, indicating that these transporters may play a role in EV production from tumors.

Human HT29 and HCT15 and murine CT26 CRC cell lines can be cultured in stem cell medium and expanded as cancer spheroids, or colonospheres, containing CRC stem cells (CRCSCs) [123]. CRC patients demonstrated an increased level of miR-146a in serum-derived EVs that appeared to correlate with decreased expression of the protein Numb; Numb expression correlates with stemness and an immunosuppressive TME [123]. Colonosphere-derived exosomes downregulated expression of 17 tumor suppressor microRNAs and upregulated expression of miR-146a. Transfer of colonosphere-derived exosomal miR-146a-5p to parental CRC cells effectively decreased expression of its target *Numb*, transferring cancer stemness phenotypes including sphere-forming capacity. Furthermore, colonosphere-derived CSCs from established cell lines secreted an increased number of bioactive EVs per cell. This can be attributed to the stem cell factor, Wnt, which activates the *β*-catenin/TCF-4 transcription factor complex and increases Rab27B expression which regulates CRCSC secretion of exosomes and the inflammatory chemokine IL-8. These are associated with stem cell functions such as self-renewal capacity and tumor growth. CSCs play a role in cancer treatment failure due to their chemoresistant properties and are increased by the physiology of tumors.

### 5.3. Microenvironmental Stress in 3D Cultures

Human ovarian cancer HeyA8 cells cultured under hypoxic conditions in stem cell medium were found to grow as spheroids and were enriched in CSCs [69]. Ovarian cancer spheroids cultured in hypoxic conditions released EVs that promoted the secretion of proinflammatory cytokines interleukin-6 (IL-6) and IL-8 and pro-angiogenic growth factor VEGF-A in bone marrow-derived mesenchymal stem cells (MSCs); these factors induced angiogenesis and the migration of low-invasive ovarian cancer cells. EVs released from the ovarian cancer spheroids in response to cisplatin elevated the levels of MMP that digests extracellular matrices, augmented migration of MSCs and increased secretion of IL-6, IL-8 and VEGF-A [124]. Thus, characterizing the effects of EVs derived from hypoxia- and chemotherapeutic drug-treated tumor cells on the TME will have important implications for cancer therapy.

Fibroblasts are part of the tumor stoma and appear to modulate tumor growth via EVs that differ in composition and effect in different environmental conditions. EVs purified from fibroblasts cultured in normoxia or hypoxia were added to CRC organoid-derived cells and only EVs from fibroblast cells cultured under hypoxic conditions increased the number of newly formed neoplastic organoids; this was not seen in cultures with EVs from monocytes or liposomes, indicating it is a fibroblast-specific function [103]. This effect also appeared to be unilateral in that treatment of fibroblasts with hypoxic CRC organoid-derived EVs did not affect the fibroblast motility or activation. This indicates the importance of tumor-induced hypoxia on fibroblast biology and EV production and the role of these EVs in tumor growth.

EVs derived from melanoma cells under different stress conditions were also found to alter 3D co-cultures of MSCs and melanoma cells (B16F1) by making their aggregates more compact [125]. The stress conditions tested in this study were cytostatic stress by doxorubicin treatment, heat stress by incubating the cells at 42 °C, or oxidative stress by treating with Ag-TiO_2_ nanoparticles [125]. The notion that normal cells under stress conditions produce EVs that contribute to malignant growth was further supported in a study of healthy breast tissue resected from patients undergoing reduction mammoplasties. When these normal cells were cultured as organoids termed mammospheres, the protein content of the EVs was affected by hormonal changes [126]. EVs derived from normal mammospheres treated with tamoxifen or estrogen contained proteins categorized as cancer-related molecules, suggesting a possible involvement of these EVs in cancer progression and metastasis [127].

### 5.4. Tumor and Stromal Cell Crosstalk in the TME

Evidence of EV-mediated crosstalk between cancer cells and tumor stromal cells has been reported. Human CRC-derived EVs (cell line SW480) have been shown to induce atypical morphology and increase proliferation, migration and invasion in primary normal human colonic MSC monolayers [128]. Treatment of 3D MSC spheroid cultures with CRC cell-derived EVs increased spheroid volume and reduced pH of the culture medium, implying increased proliferation and extracellular acidification. MSC spheroids treated with human metastatic CRC SW620 cell-derived EVs exhibited an increase in proliferation and acidification. Primary CRC cell EVs increased the expression of the important clinical CRC marker carcinoembryonic antigen (CEA) in MSC spheroids and a redistribution of the surrogate tumor marker vacuolar H^+^-ATPase (V-ATPase) pH regulator protein from the cytosol to the plasma membrane. Expression of CEA and plasma membrane V-ATPase increased further upon MSC spheroid treatment with metastatic CRC cell EVs [128]. Primary human colon cancer MSCs formed larger spheroids sooner than healthy colonic MSCs in 3D cultures and exhibited increased proliferation and expression of CEA and V-ATPase when treated with primary or metastatic CRC cell EVs. In this study, colon cancer MSCs formed an umbilicated 3D morphology, characteristic of the necrotic center typical in a tumor mass in vivo [128].

EV crosstalk has been reported in a variety of different cancers, supporting the notion that cellular communication in the TME is multidimensional. The effects of human prostate cancer Du145 cell-derived EVs on MSC differentiation were also investigated using spheroids consisting of cancer cells alone or a mix of cancer cells and bone marrow-derived mesenchymal stem cells (BM-MSCs) embedded in Matrigel. Spheroids comprised of BM-MSCs and MVB docking protein Rab27a-knockdown Du145 cells showed a significantly delayed extra-spheroidal cell outgrowth compared to that of spheroids consisting of control Du145 cells and BM-MSCs. Purified EVs from Du145 cells skewed the differentiation of BM-MSCs towards *α*-smooth muscle actin (*α*-SMA)-expressing myofibroblasts, which secrete high levels of VEGF-A, hepatocyte growth factor (HGF), MMP1, MMP3 and MMP13 [129]. The effect of MSC-derived EVs on breast cancer spheroids cultured in a type I collagen gel have also been characterized [130]. MSC-derived EVs induced breast cancer spheroids to become more compacted. Co-culturing MSC spheroids with breast cancer spheroids upregulated the expression of epithelial cell markers, E-cadherin and keratin19, with a decreased expression level of mesenchymal cell markers, vimentin and JUP, in the breast cancer spheroids. Thus, MSC-derived EVs promote mesenchymal to epithelial transition (MET), characteristic of metastatic cancer cells colonizing their secondary microenvironment [131].

There is evidence of tumors communicating with immune cells via EVs. Human prostate cancer PC3 cells expressing CD63-GFP mixed with human peripheral blood mononuclear cells (PBMCs) seeded at high density in non-adhesive plates were found to form 3D heterotypic spheroids [72]. The transfer of GFP-labeled EVs from PC3-CD63-GFP cells to the lymphocytes was analyzed by flow cytometry and fluorescence microscopy. The GFP-labeled EVs interacted with a large fraction of B cells, although the majority of EVs were not internalized by B cells and appeared to be bound at the cell surface. T cell subsets, CD3^+^ and CD8^+^ T cells, differed in their ability to interact with the GFP-labeled EVs and a fraction of EVs were internalized in CD3^+^ T cells via macropinocytosis [72]. EVs secreted from epithelial ovarian cancer (EOC) tissue explants, ovarian cancer cell lines (OVCAR-3 and SKOV-3) and ascitic fluid from EOC patients were analyzed for their role in immunosuppression via two pathways involved in anticancer immunity, NKG2D receptor-ligand and DNAM-1-poliovirus receptor (PVR)/nectin-2 [132]. Both pathways are involved in the elimination of tumor cells by natural killer (NK) and natural killer T cells [133]. Compared to healthy ovarian tissue, EOC tissue explants and cancer cell lines showed higher levels of ligands for the NKG2D pathway, while DNAM-1 ligands are more seldom expressed and not associated with the EV membrane surface. Consequently, the NKG2D-ligand-bearing EOC EVs significantly down-regulated the NKG2D receptor expression in PBMCs, while the DNAM-1 receptor was unaffected. Thus, 3D spheroids are a model for characterizing crosstalk between cancer cells and stromal cells via EVs within the TME, providing crucial information on cell and EV behavior within a 3D matrix.

## 6. Modeling How Tumors Reach Out to Adjacent and Distant Non-Malignant Tissues via EVs

Cancer cells are constantly secreting EVs to adjacent surrounding tissues and into the circulation that interact with distant non-malignant host cells, generating a systemic tumor-host “macroenvironment” [52,134,135,136]. These tumor-derived EVs may send aberrant signals inducing a pro-tumor phenotype in adjacent surrounding cells, recruit immune and stromal cells from the bone marrow, induce tumor-associated phenotype in immune cells and fibroblasts, or aid in forming potential sites of metastasis. 3D models including organ-on-a-chip cultures and zebrafish allografts can be used to track and characterize the effects of tumor-derived EV interactions with adjacent non-malignant host cells and participate in the formation of a pre-metastatic niche.

### 6.1. Affecting the Normal Surrounding Tissue—Systemic Pathologies from Cancer EVs

The proneoplastic effects of esophageal adenocarcinoma cell-derived EVs on normal gastric epithelial organoids (gastroids) have been delineated. Esophageal adenocarcinoma cell line-derived EVs were labeled with PKH67 and co-cultured with gastroids derived from normal human gastric tissues cultured in Matrigel with gastrin and nicotinamide [77]. Cancer cell-derived EVs co-cultured with normal gastroids promoted proliferation and suppressed apoptosis, via oncogenic noncoding RNAs, miR-25 and miR-210, inducing a neoplastic phenotype. This study implies that cancer cells in the TME may secrete EVs that aberrantly signal to surrounding normal stromal and immune cells, promoting a pro-tumorigenic phenotype. EVs from cancer cells may also communicate to other areas of the body.

Cancers often lead to cachexia, a syndrome linked to weight loss and fatigue and the zinc transporter, ZIP4, has been shown to be involved in the induction of cachexia from pancreatic tumors [137]. Serum from pancreatic cancer spheroids and 2D cultures contained HSP70 and HSP90 and induced negative effects on muscle cells grown in culture, such as activation of p38MAPK, which were decreased when ZIP4 was knocked down. EVs from pancreatic cancer cells were enriched in HSP70 and HSP90, induced activation of p38MAPK in myotubes and produced more EVs when ZIP4 was present. It was found that ZIP4 induced EV production by stimulating CREB-regulated RAB27B expression, indicating that ZIP4 regulates EV release to promote cachexia [137].

### 6.2. Predisposition to Metastasis—EVs Cultivate the Premetastatic Niche

Following detachment from the primary tumor and intravasation, circulating tumor cells (CTCs) must extravasate at a distant site, survive and later thrive in a foreign tissue microenvironment to complete the metastatic cascade [138]. The premetastatic niche, a supportive microenvironment or “nest” at a site of future metastasis, is often formed by tumor-derived EVs that exit the leaky vasculature of the TME, circulate in the blood stream and lodge in distant sites in non-malignant host tissue creating a “welcoming” environment for CTCs [139]. Tumor-derived EVs and CTCs both exhibit organotropism mediated by surface receptors such as the chemokine CXCL12/CXCR4 in breast cancer metastasis, integrin *α*_6_*β*_4_ and *α*_6_*β*_1_ in lung metastasis and integrin *α*_v_*β*_5_ in liver metastasis [140,141]. Novel EV imaging methods developed in 3D models allow for better tracking of EVs in the metastatic process. One such method is the to use bioluminescence resonance energy transfer (BRET), which allows for both fluorescence and bioluminescence imaging. An example is the PalmGRET reporter utilizing GFP and Nanoluc bioluminescence reporter genes fused to a palmitoylation signal peptide, allowing the reporter to be trafficked to the cell membrane and expressed in EVs [84]. Mouse hepatocellular carcinoma cells (HCA1) stably expressing PalmGRET EVs were injected into mice and had greater distribution to liver and lungs. Knocking down lung tropism-related proteins reduced EV uptake in the lungs, indicating that the HCA1 EVs may be targeted to the lungs to promote metastasis [84].

Murine models have provided crucial information about the formation of a premetastatic niche [142,143,144]. Distribution of fluorescently labeled EVs within a tissue can be visualized in real time via confocal laser scanning microscopy (CLSM) or by histological analysis of resected tissues [143,145]. EV pharmacokinetics are often tracked in vivo via BLI using EV markers which allow EV biodistribution to be tracked over time [36,131]. This method lacks sensitivity and requires secondary injection of substrate but is nonetheless informative. Radioisotope-labeled EVs can be detected deeper within a tissue by single photon emission computed tomography (SPECT) or positron emission tomography (PET) [146,147]. EVs containing iron nanoparticles can also be imaged via magnetic resonance imaging (MRI) or magnetic particle imaging (MPI), providing a better signal-to-noise ratio [37,148,149]. However, in vivo imaging methods are limited in their ability to track EVs and contents following the initial distribution and subsequent elimination phase and fail to track EVs that have undergone transcytosis [150]. For this reason, the mechanism by which the secondary organ microenvironment is affected by tumor-derived EVs largely remains to be elucidated. The utilization of organ-on-a-chip models and zebrafish allograft models can surpass the challenges in cost, time and capabilities of EV tracking in murine models. In addition, these models may potentially lead to new opportunities in personalized diagnostic and therapeutic strategies for metastasis.

**Organ-on-a-chip** models are bioinspired microfluidic chips made of transparent polymeric constructs containing channels that are lined by living cells to mimic key organ activities and functions [151,152,153]. Organ-on-chips are designed to model certain tissues, organ units, or cells cultured in microchannels [154]. Perfusion is advantageous in studying metastasis due to limited crosstalk between cell culture compartments, better representing the continuous unidirectional flow of in vivo circulation [155]. Furthermore, organ-on-chip approaches enable control of local concentrations of molecular signals such as growth factors, chemokines and hormones, allowing perturbation and study of the interactions between tumor-derived EVs and organ-specific cells [156].

One example is the all-human 3D liver microphysiological system (MPS)—a perfusion 3D culture of multiple liver cell types, primary hepatic tissue and/or organoids that is shown to be reproducible and more clinically relevant than spheroids and sandwich cultures with regards to drug toxicity, metabolism and intracellular accumulation [157]. The ability of this liver MPS to maintain primary hepatic cells long-term, allows for characterization of the bidirectional EV interactions between metastatic breast cancer and normal liver cells. Injection of breast cancer cell-derived EVs into the liver MPS confirmed previous findings that EVs contributed to formation of a pre-metastatic niche and promoted the homing of cancer cells in the liver. The response of the hepatocytes in the liver MPS and 2D co-cultures was to secrete exosomes, containing anti-oncogenic microRNAs, that suppressed proliferation and invasion of metastasized breast cancer cells [158]. The interactions between normal and malignant cells in the MPS model system demonstrated the intercellular interactions that can be modeled and reveal competition between the two cell types, one trying to build a niche and the other trying to resist.

Organ-on-a-chip models can recapitulate cellular interactions and have consequently contributed to our understanding of the organotropism of EVs and CTCs in the formation of the premetastatic niche and subsequent tumor cell homing [154,159,160]. A 3D microfluidic chip was recently developed to mimic breast cancer liver metastasis and elucidate the role of primary tumor-derived EVs in the formation of the premetastatic niche [161]. This 3D human liver-chip was comprised of a co-culture of hepatocytes with liver fibroblasts and sinusoidal endothelial cells in multiple physiologically relevant layers that recapitulated the liver microenvironment and sustained cell viability, albumin secretion and urea synthesis. Flow injection of tumor-derived EVs through the space mimicking the vessel lumen downregulated tight junctions in endothelial cells, induced the transdifferentiation of endothelial cells toward a mesenchymal phenotype (EndMT) and increased expression of cancer-associated fibroblast markers and the tumor marker *α*-fetoprotein. Increased endothelial cell uptake of DiD-stained tumor-derived EVs increased subsequent breast cancer cell adhesion to liver endothelial cells and invasion into the liver microenvironment. Tumor-derived EVs contained TGF-*β*1 which upregulated fibronectin in endothelial cells, leading to increased breast cancer cell adhesion. EV-induced increases in breast cancer cell adhesion to liver endothelial cells was confirmed in liver-chips containing primary human liver cell types and using EVs isolated from plasma of breast cancer patients with liver metastases [161]. Such analyses of molecular signaling in creation of cellular niches is not possible in animal models and help define the role of organ-on-chip models in biomedicine.

A biomimetic multiorgan liver–kidney-on-a-chip was also recently developed with two chambers containing viable precision-cut tissue slices (PTSs) obtained from rat tissue explants [162]. PTSs mimic the multicellular characteristics of organs and generate a stronger chemokine gradient than monolayer cultures. In addition, because each rat can provide 8-10 PTSs, these tissue section-based organ-on-a-chip models save time and expense compared to animal models. Human breast cancer MDA-MB-231 and MCF7 cell-derived EVs perfused through the microsystem showed strong liver tropism. This was mainly because liver explants generated a 2-fold higher CXCL12 gradient than kidney explants, strongly attracting the CXCR4-expressing cancer cells and EVs. Furthermore, EVs from highly metastatic MDA-MB-231 had higher liver tropism than those from the low-metastatic line MCF7. Tissue explants cultured in the microfluidic chip generated a 1.5-fold increased EV localization to liver tissue sections as compared to tissue explants in the chemotaxis assay using Transwell chambers, similar to what was observed in mouse models [162].

To track tumor-derived EVs as they cross the blood–brain barrier (BBB), Morad et al. designed a microfluidic organ-on-a-chip model of the BBB [150]. This BBB-on-a-chip consists of a basal vascular channel lined by iPSC-derived human BMECs separated by a porous membrane coated with ECM proteins from an apical parenchymal (abluminal) channel containing primary human astrocytes and pericytes [163]. Human breast cancer MDA-MB-231 cells were transduced with lentiviral vectors to express palmitoylated tdTomato or membrane-bound Gluc. Palmitoylated tdTomato-expressing tumor EVs were administered into the lumen of the vascular channels and fluorescent signal was detected inside astrocytes in the abluminal channel after 3 hours and increased over time, without increasing permeability of the BBB to 10 kDa and 70 kDa dextran. The detection of intact fluorescent-stained EVs in endothelial cells as well as astrocytes during tumor EV perfusion through the vascular channel lumen of the BBB-on-a-chip model elucidates transcytosis as a mechanism of tumor EV interaction with BMECs under flow conditions [150]. Tumor EV treatment-induced BMECs to decrease expression of the late endosomal marker rab7, decreased transfer to lysosomes and increasing rate of EV uptake. These EV interactions with endothelial monolayer can be compared to a static model of the BBB, where Gluc-expressing tumor EVs were added to the top chamber of a Transwell plate with a monolayer of endothelial cells. Uniquely, Gluc does not require ATP, rendering Gluc expressing EVs capable of producing luminescent signal upon addition of its substrate coelenterazine. The luciferase assay revealed that the luminescent signal from tumor EVs was detectable in the bottom chamber of the Transwells after two hours. This signal was decreased by incubating cells in cooler temperatures and by inhibiting endocytosis via Dynasore, demonstrating that EVs are actively transported across the brain endothelial monolayer [150]. Although the Transwell model provided valuable preliminary information, static incubation of endothelial cells with tumor EVs cannot physiologically mimic the interaction of EVs with vascular cells under the perfusion pressure of the in vivo circulatory system. The BBB-on-a-chip is, thus, a significantly effective model for better understanding and tracing of EV interactions with the complex human brain vasculature.

**Zebrafish** models have been developed to visualize dynamic EV and cell movement inside the TME, in the circulation and during metastasis (see Figure 1). There have been many evolutionarily conserved genetic and molecular pathways in humans and fish that drive cancer progression [164]. Zebrafish embryos are advantageous for studying EV movement and interactions in circulation due to the similarity of the vasculature to that of humans and the model’s transparency that allows for live imaging [165]. Additionally, zebrafish embryos are easily genetically modified and have a high fecundity allowing for high-throughput screens [166].

High resolution confocal microscopy can demonstrate human cancer cell intravasation, interactions with zebrafish vasculature and extravasation in real time [167,168,169]. Because zebrafish xenografts develop a mature BBB at three days post fertilization, they have also proven an effective model in representing the tight-junction based permeability of the human BBB and its effects on brain metastasis [165]. Metastatic breast cancer MDA-MB-231 brain-seeking cell-secreted EVs have been visualized in the zebrafish embryo passing the in vivo BBB via transcytosis without compromising the junctional permeability of the BBB and subsequently being taken up by cells in the brain parenchyma one-hour post EV injection [150]. However, zebrafish xenograft models are limited in their ability to accurately represent clinical conditions of the TME and metastasis because the cells of the TME are interacting with stromal and immune cells of a different species and class and human tumor xenografts are implanted in a non-physiologic site, misrepresenting systemic signaling to the TME. In addition, the adaptive immune system is not functional in this model, meaning that results obtained using embryo may not always reproduce the behavior of cancers in a fully immunocompetent host [170].

P53 deficient zebrafish with a BRAF mutation (V600E) have been discovered to induce melanoma in zebrafish, providing a tumor allograft model overcoming many of the disadvantages of xenografts [171,172]. The zebrafish melanoma Zmel1 cell line secretes EVs similar in protein content to human and murine melanoma cell line EVs, showing clinical relevance [164]. Prior to injection into the transparent zebrafish embryo, tumor cells and EVs were labeled with Syntenin2-GFP or dyed with MemBright—a bright cyanine-based fluorescent membrane probe [173]. Live confocal imaging allowed simultaneous visualization of tumor cell and EV behavior in the TME, blood circulation and metastasis [164]. After intravenous injection, Zmel1-derived EVs were tracked flowing through circulation, rolling and arresting on the endothelium surface. Within minutes, tumor EVs were taken up mostly by endothelial cells and patrolling macrophages. Interestingly, these cells also took up control beads and fibroblast cell-derived EVs to a lesser extent, suggesting that in vivo EV uptake can occur by both specific and nonspecific mechanisms. Within two days, Zmel1 cells injected into circulation extravasated, transferring EVs to locally residing macrophages. Tumor cell injection into the yolk region formed an in vivo TME, allowing for tracking of in vivo-grown tumor EVs prior to metastasis. Tumor EV uptake significantly reduced macrophage motility and pro-inflammatory TNF-*α* secretion. Notably, tumor EVs primed pre-metastatic niche formation upon injection into zebrafish embryos, increasing metastatic outgrowth and invasiveness upon subsequent tumor cell injection [164]. Thus, zebrafish embryos are a novel model for live cell imaging and EV tracking during all stages of in vivo metastasis.

**Table 1:** Key advantages and limitations of the currently available 3D culture models for studying EVs.

TME Model	Common Methods of EV Imaging	Advantages	Limitations
2D cell monolayers	▪ Lipophilic dye (e.g., PKH, DiR, or DiI) [36] ▪ Fluorescent protein labeling [36] ▪ Bioluminescent protein labeling [82] ▪ Raman Imaging [89]	▪ Quick ▪ High throughput ▪ Cost-effective	▪ Unrepresentative protein/gene expression [21,32] ▪ Cell-cell interactions confined to 2D plane ▪ Fewer cell-ECM interactions
Small mammalian animal models	▪ Lipophilic dye [143] ▪ Fluorescent protein labeling [145] ▪ Bioluminescent protein labeling [36,131] ▪ Intravital fluorescence microscopy [36] ▪ Nuclear imaging i.e., SPECT, PET [146,147] ▪ MRI, MPI [36,37]	▪ 3D cellular interactions within TME ▪ TME crosstalk with systemic signals	▪ Low clinical success rate [44,45] ▪ Non-human cell types ▪ Cost-intensive ▪ Time-consuming ▪ Difficult to track EVs long-term [40]
Cancer spheroids	▪ Lipophilic dye [66,110,119,123] ▪ Fluorescent protein labeling [109,118]	▪ EV size, cargo and biogenesis representative of patient plasma EVs [64,65] ▪ Spatial and physical aspects of TME (e.g., 3D cell-cell and/or cell-ECM interactions) [46] ▪ Relatively simple set up for cancer cell culture and co-culture systems ▪ CSC production [66]	▪ Limited cell types present in a tumor
Stem cell-derived organoids	▪ Lipophilic dye [77]	▪ Representative EV size, cargo and biogenesis of patient plasma EVs [62] ▪ Self-organized by self-renewal [78] ▪ Heterogeneity of cell types and stemness gradient present in an organ and EVs	▪ Higher cost for growth factors ▪ Long-term culture required for stem cell differentiation
Organ-on-a-chip	▪ Lipophilic dye [158,162] ▪ Fluorescent protein labeling [150]	▪ Spaciotemporal control of biochemical signals [156] ▪ Unidirectional perfusion vascular systems [155] ▪ Steady cell metabolism for over time [157] ▪ Long-term culture of normal cells and tissues	▪ Lacking models of the immune system in TME [159] ▪ More laborious ▪ Cost of hardware e.g., chips, microfluidic pumps
Tissue explants	▪ Label free multimodal imaging [91,92]	▪ Preserves original tissue architecture [79] ▪ Physiologically relevant [79]	▪ Short-term cultures [79] ▪ Cost-intensive [79] ▪ Lower feasibility
Zebrafish	▪ Lipophilic dye (e.g., MemBright) [164] ▪ Fluorescent protein labeling [164]	▪ Similar complex vascular systems to humans ▪ Transparency of embryos for live cell and EV imaging ▪ High throughput due to ease of genetic modifications and high fecundity	▪ Xenograft models misrepresent systemic signaling to the TME due to xenograft implantations in non-physiologic site ▪ Differences in host niche and environmental factors in xenografts

## 7. Future Perspectives

**Figure 3:**
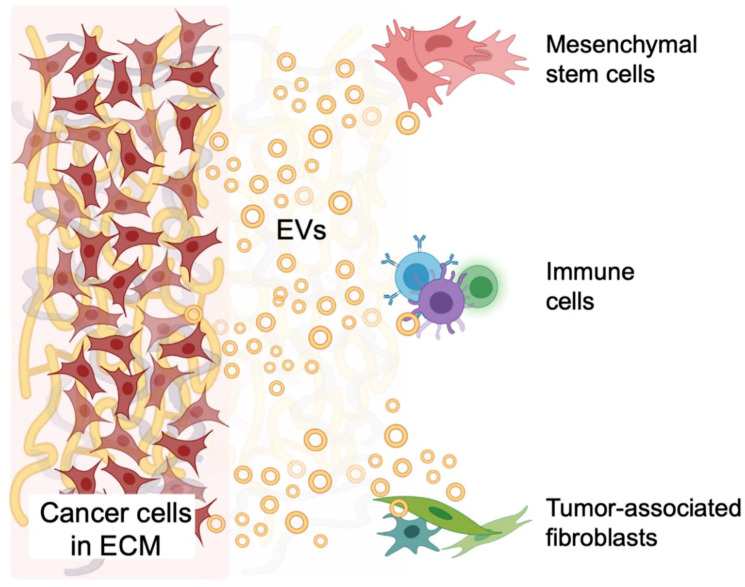
Schematic illustration of how 3D extracellular matrix (ECM) within the tumor microenvironment (TME) can affect the phenotype of tumor cells and biogenesis of extracellular vesicles (EVs) and how EVs interact with ECM and subsequently signal to tumor-associated stromal cells such as mesenchymal stem cells, immune cells and tumor-associated fibroblasts. Created with BioRender.com (accessed on 3 April 2021).

Much progress has been made in understanding EV communication within the 3D TME, but future investigations are needed to complete our understanding (see Box 2). Current challenges of 3D models include the cost associated with more complicated models such stem cell-derived organoids, as well time and cost required for adaption of organ-on-a-chip microfluidic pumps and zebrafish breeding facilities. Further research is needed to create and characterize new biomaterials that better mimic the ECM in 3D cultures. Due to the effects of scaffolding on tumor cell behavior and EV biogenesis in 3D spheroid models, there is a need to investigate the signaling effects of EVs derived from tumor cells cultured in 3D scaffold on stromal cells, as depicted in Figure 3. Novel EV imaging methods, such as label-free multimodal multiphoton techniques, can be applied to EV imaging and tracking in 3D cultures, as in vitro models allow for studying of EV uptake that cannot be done in vivo. Monitoring different EV populations and characterizing their signaling effects within a 3D model will aid in our understanding of how EVs from different cell types are involved in maintenance of the complex TME. Studies can include EV interactions through 3D vasculature in tumor-on-chip, including “maintained biochemical gradients, perfused angiogenic sprouts and control over the spatial arrangement of multiple cell types” [174], EV organotropism in organ-on-chip, spheroids and organoids in organ-on-chip and the application of novel 2D imaging methods to 3D models.

The scientific interest in the use of EVs in therapy and diagnostics has also bloomed in interdisciplinary research areas such as molecular communication (MC). Researchers working in this field use the mathematical tools and metrics applied in information and communication technology (ICT) to build computational models which characterize the functions of biological signaling systems [175,176]. The EV-based MC models complement models from computational biology and create a new in-silico approach in analyzing EV-mediated signaling. Input/output analyses are envisioned to predict EV-associated cell response to the chemical, electrical or mechanical factors, replacing in vitro experiments [177]. Metrics such as information channel capacity, error probability, throughput and latency can further provide an in-depth analysis of EV signaling pathways and, for example, optimize EV dosage and the efficacy of the EV-based therapy [178].

In the context of EV-mediated signaling in the TME, EVs are considered as physical carriers of information exchanged between cells, that together with the TME, create a communication system. Future investigations in the MC field are needed to fully model all elements of EV-mediated signaling in the 3D TME from the perspective of information, communication and network theories. For example, the secretion of EVs needs to be characterized and modeled in various cell types, as well as the uptake by the recipient cells where the models on viral entry may provide details to look for clues. The open research questions also include 3D channel models on the EV advection (due to the interstitial fluids) and diffusion (due to the concentration gradient) in the TME. The communication channel models will relate the received concentration of EVs at the recipient cell to the secreted concentration of EVs at the donor cell as a probability mass/density function to capture uncertainties imposed by the noisy processes inside the TME. Such processes are caused by the TME non-homogeneity and properties such as volume fraction and tortuosity, all contributing to the anisotropic EV propagation. The volume fraction defines the percentage of the total TME volume accessible to EVs, whereas the tortuosity describes the average hindrance of a medium relative to an obstacle-free medium. Hindrance results in an effective diffusion that is decreased compared with the free diffusion coefficient of EVs. In addition, morphological characteristics of EVs themselves, including size, density, shape, lipid or protein composition increase the number of variables in modeling.

Box 2 **Outstanding questions.**

- Can we characterize animal models regarding their clinical relevance to human disease and use this to better inform the development of our culture correlates?
- How dramatically do EV imaging methods affect the signaling fate of EVs in monolayers, 3D organ models and in vivo?
- How do scaffold types affect signaling of tumor cell-derived EVs to stromal cells?
- How can we trace EVs undergoing transcytosis long-term and determine its relevance in human disease?
- Can we model organotropism in organ-on-a-chip cultures for various cancer types, for immune cells and in organogenesis?

## 8. Conclusions

3D cultures that mimic the human TME can better replicate the tumor stromal environment and EV biogenesis and uptake than can 2D cultures, which lack the 3D tumor stromal environment and mouse models, which may exhibit lower clinical translatability and challenges in tracking EVs long-term. Cells cultured in different scaffold types in 3D spheroids often exhibit cell phenotypes and EV biogenesis, size and cargo that are more representative of tissues and organs. Simple 3D scaffold-free spheroid cultures have elucidated many aspects of EV signaling in the 3D TME, including EV-mediated tumor–stromal crosstalk and the role of CSCs in tumorigenicity and chemoresistance. Stem cell-derived complex 3D organoid cultures produce more heterogenous tumor cell and EV populations. Tissue explants, less feasible and more costly, contain a fully heterogeneous population of cells and EVs and novel techniques have been developed to localize EVs within tissues including label-free multiphoton imaging of EVs. Organ-on-a-chip cultures have demonstrated EVs exhibit organotropism resembling cells of origin and aid in the formation of the premetastatic niche. The zebrafish allograft model of melanoma has recently led to the capability of simultaneously imaging tumor cells and EVs at every stage in metastasis. In the field of molecular communications, EV secretion and uptake have recently begun to be modeled in tumor cells according to factors such as EV morphology, density and composition. Despite increasing clinical relevance, 3D models have not been incorporated into widespread use in the field of EV research and there is additional development that is needed to represent the full complexity of intact tissues and organs. Collaborations between EV biologists and engineers of 3D tissue models will elucidate physiologically relevant EV-mediated signaling effects within the TME.

## Abbreviations

TME	tumor microenvironment
EV	extracellular vesicle
ECM	extracellular matrix
MV	microvesicle
MVB	multivesicular body
ILV	intraluminal vesicle
ESCRT	endosomal complexes required for transport
MISEV	minimal information for studies of extracellular vesicles
CSC	cancer stem cell
iPSC	induced pluripotent stem cells
ESC	embryonic stem cells
PDE	patient-derived tissue explants
BBB	blood–brain barrier
BLI	bioluminescent imaging
MSC	mesenchymal stromal cell, mesenchymal stem cell
CRC	colorectal carcinoma
GBM	glioblastoma multiforme
